# Comparison of KF-Based Vehicle Sideslip Estimation Logics with Increasing Complexity for a Passenger Car

**DOI:** 10.3390/s24154846

**Published:** 2024-07-25

**Authors:** Lorenzo Ponticelli, Mario Barbaro, Geraldino Mandragora, Gianluca Pagano, Gonçalo Sousa Torres

**Affiliations:** Industrial Engineering Department, University of Naples ”Federico II”, Via Claudio 21, 80125 Naples, Italy; mario.barbaro@unina.it (M.B.); geraldino.mandragora@unina.it (G.M.); gianluca.pagano3@unina.it (G.P.); goncalo.sousatorres@unina.it (G.S.T.)

**Keywords:** vehicle state estimation, vehicle dynamics, extended Kalman filter, unscented Kalman filter, sideslip angle, virtual sensing

## Abstract

Nowadays, control is pervasive in vehicles, and a full and accurate knowledge of vehicle states is crucial to guarantee safety levels and support the development of Advanced Driver-Assistance Systems (ADASs). In this scenario, real-time monitoring of the vehicle sideslip angle becomes fundamental, and various virtual sensing techniques based on both vehicle dynamics models and data-driven methods are widely presented in the literature. Given the need for on-board embedded device solutions in autonomous vehicles, it is mandatory to find the correct balance between estimation accuracy and the computational burden required. This work mainly presents different physical KF-based methodologies and proposes both mathematical and graphical analysis to explore the effectiveness of these solutions, all employing equal tire and vehicle simplified models. For this purpose, results are compared with accurate sensor acquisition provided by the on-track campaign on passenger vehicles; moreover, to truthfully represent the possibility of using such virtual sensing techniques in real-world scenarios, the vehicle is also equipped with low-end sensors that provide information to all the employed observers.

## 1. Introduction

The automotive industry is continuously evolving toward an increasing level of vehicle automation in accordance with the necessity of improving passenger safety and comfort to reduce the number of road accidents. To answer this crucial social challenge, one of the priority areas involved in the research field of vehicle dynamics concerns the design and development of ADASs (Advanced Driving Assistance Systems) that optimize vehicle interaction with humans and with the external environment (road, weather conditions, tire conditions) [1,2,3,4,5,6,7,8,9,10,11].

In this scenario, on one hand, accurate knowledge of the vehicle’s instantaneous state is crucial to optimize the control logic [12,13,14,15,16,17,18]; on the other hand, some physical variables can be measured only by employing expensive high-end sensors, making it hard to ensure the large-scale availability of the designed system. In particular, the knowledge of the vehicle sideslip angle is essential in vehicle lateral dynamic applications, such as stability control and trajectory planning for autonomous vehicles [19,20]. However, its on-board measurement requires high-cost vehicle-sensors equipment. Therefore, to extend the application to a less customized scenario, virtual sensing techniques are adopted to allow the estimation of the sideslip angle only employing low-end sensors, which are suitable for mass-production vehicles [21,22,23,24,25,26,27,28,29,30].

Three main sideslip angle estimation approaches are currently available in the literature: kinematic-based, tire model-based, and data-driven approaches.

Data-driven approaches are usually designed to achieve accurate results in both linear and nonlinear vehicle operating conditions, employing AI methodologies to capture the vehicle state toward the measured available signals [31,32,33,34,35]. The main disadvantage of this approach is related to the necessity of a large and diversified dataset covering the entire vehicle operating range in order to perform the tuning of the algorithm without incurring the overfitting risk.

Instead, the kinematic estimator only includes kinematic relationships employing the real-time measurement of accelerations, yaw rate, and wheel linear velocities. In this case, the calibration routine of the tire and vehicle model is not required, and the estimation process is robust toward tire parameter variations [36,37,38,39]. However, the estimator becomes unobservable when the vehicle yaw rate approaches zero and the estimation accuracy is strongly affected by the sensors’ noise, resulting in cumulated errors due to the integration process.

The current paper focuses on the dynamic tire model-based approach, which involves the implementation of a dynamic model adopting the vehicle equilibrium equations and employing a tire model to calculate the forces acting at the ground plane, which are dependent on the instantaneous kinematic state of the vehicle [40,41,42,43,44,45,46]. Although this approach is more accurate and less corrupted by noise with respect to the previous one, it is significantly sensitive to the reliability of the identified tire parameters and to the capability of the estimator to capture the tire dynamics both in the linear and nonlinear regions [47,48,49,50].

In this scenario, the model selection plays a crucial role in optimizing the estimator design: in the current work, the 3-dof single-track model has been adopted. Although this formulation is less accurate than others discussed in the literature, which employ a multibody modeling approach [51,52,53,54,55], it requires a simpler parametrization ruotine. Moreover, since the vehicle model is integrated within an observer architecture [56,57,58,59,60], the formulation has to satisfy the observability condition: the available measurements have to uniquely define the state to optimize the correction based on the instantaneously acquired signals. Using the single-track approach makes it easier to respect this criterion also employing a minimum number of measurements, since the model equations are easily invertible.

In this regard, techniques based on the Kalman filter logic are frequently employed to build the mentioned observer architecture, including the model equations and the measurement corrections. These algorithms could be designed with differing formulations, depending on the required level of complexity. In those conditions where the vehicle dynamics could be simulated through a linear formulation, the extended Kalman filter consists of the most employed solution to accurately perform the estimation without increasing the computational cost of the estimator [61,62,63]. However, when the vehicle moves toward strongly nonlinear conditions, the estimation errors due to linearized formulation become non-negligible, leading to the necessity of employing more accurate and complex estimation logic [64,65,66].

The paper proposes qualitative and quantitative comparisons to evaluate the performance of differing Kalman filter-based methodologies, characterized by increasing complexity, to estimate the vehicle sideslip angle, with the aim of maximizing the balance between accuracy and computational cost, also accounting for the deviation of the vehicle behavior from the nonlinear range. A unique single-track model has been designed and calibrated through an identification routine based on experimental data, and the estimation process has been performed using a fixed set of on-board measurements, which are commonly available on mass-production vehicles. Compared to previous existing works [67], the analysis has been performed by dividing the experimental data in differing driving conditions to verify the improvement provided by highly nonlinear formulations in each explored vehicle operating range compared with the simplest EKF implementation.

The experimental campaign also provided accurate measurements for the validation of the estimator, allowing the comparison of the estimation results with an accurate experimental target. Moreover, since the aim is to analyze the results both in linear and nonlinear working conditions, the data collection has been carried out by exploring vehicle behavior in differing maneuvers.

The paper is structured as follows: Section 2 provides an overview of the various Kalman filter typologies available in the current state of the art, including an accurate analytical description of their respective architecture. Section 3 describes the model-based approach employed within the estimation procedure, including the mathematical formulation of the vehicle model, tire model, and measurement update equations. In Section 4, the experimental activity required for the tire model characterization and the filters’ validation is reported, both describing the vehicle-sensors equipment and specifying the signals used in the estimation logic. The results are presented in Section 5 and discussed in the conclusions in Section 6.

## 2. KF-Based State Estimation Techniques

Model-based estimation design deals with compromising the output’s accuracy and the level of complexity. Various kinds of observers are presented in the literature; this work is mainly based on the Kalman filter (KF) [68]. The original formulation was thought to be applied to linear systems; however, it is well known that real systems are, in general, hardly nonlinear. To better understand these kinds of filters, it is mandatory to provide the reader with some general context to this approach. In 1960, R.E. Kalman defined a recursive solution to the discrete-data linear-filtering problem. The presented approach involves the use of a set of mathematical relations that generates an estimate of the process’s state in a way that minimizes the mean of the squared error. Linear processes can be formulated in discrete-time form through process and measurement equations. The general formulation is now introduced (Equations (1) and (2)):

(1) $$x_{k}=A\;x_{k-1}+B\;u_{k-1}+W\;w_{k-1}$$

(2) $$z_{k}=H\;x_{k}+V\;v_{k}$$

where

- $x_{k}$ is the state vector at time step *k*.
- $u_{k-1}$ is the input vector at time step k−1.
- *A* is the matrix that dynamically describes the state’s evolution.
- *B* is the matrix that correlates the input and state variables, which is also known as the control matrix.
- *H* is the measurement matrix.
- *W* and *V* are the process and measurement noise matrices.

**Figure 1:**
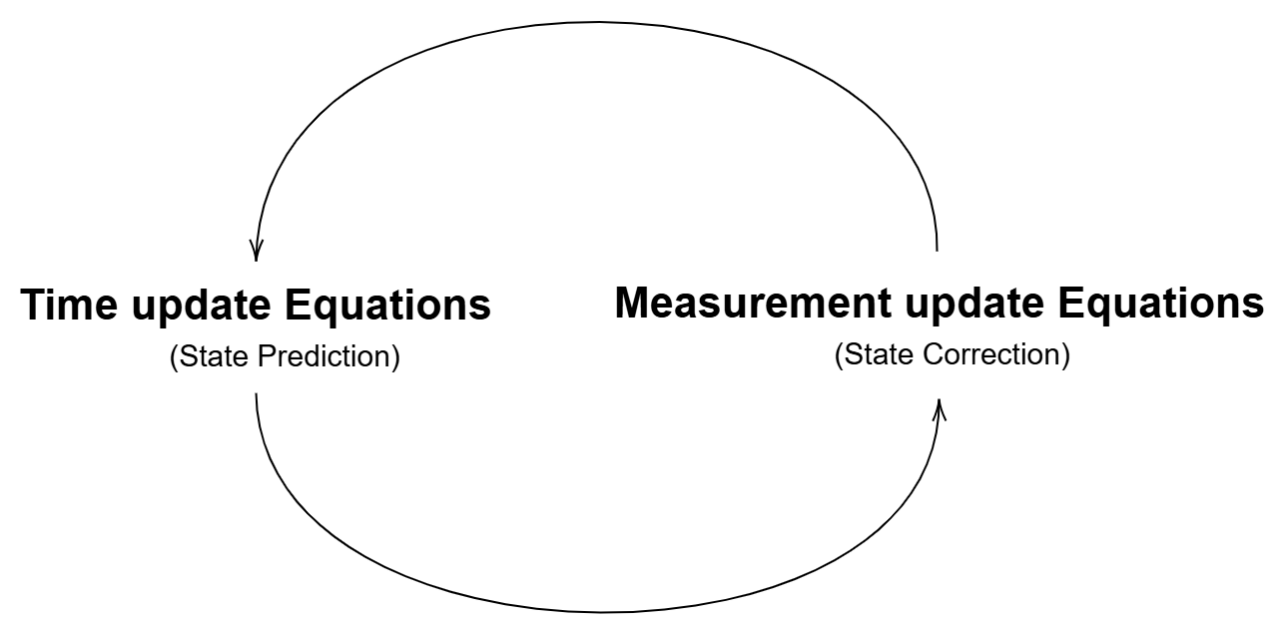
The Kalman filter cycle.

In general, $x_{k}$ is a column vector with N elements depending on the complexity of the system, while $w_{k}$ and $v_{k}$ represent the process and measurement noise with *Q* and *R* being the correspondent covariance matrices. The set of equations of the KF-filter is divided into time update equations and measurement update equations (Figure 1).

In discrete KF filter logic, time update equations (Equations (3) and (4)) provide the evolution of the system a priori, and they rely only on the system model:

(3) $${\hat{x}}_{k}^{-}=A\;{\hat{x}}_{k-1}+B\;u_{k-1}$$

(4) $$P_{k}^{-}=A\;P_{k-1}\;A^{T}+W\;Q\;W^{T}$$

where ${\hat{x}}_{k}^{-}$ indicates the a priori estimated state at time step k, $P_{k-1}$ the state covariance at time step (k−1), $P_{k}^{-}$ the a priori state covariance at time step k. On the other hand, the measurement update equations (Equations (5)–(7)) allow to correct the a priori estimation based on chosen measurement, hence providing the a posteriori estimation of the state:

(5) $$K_{k}=P_{k}^{-}\;H^{T}{\left(H\;P_{k}^{-}\;H^{T}+V\;R\;V^{T}\right)}^{-1}$$

(6) $${\hat{x}}_{k}={\hat{x}}_{k}^{-}+K_{k}\left(z_{k}-H\;{\hat{x}}_{k}^{-}\right)$$

(7) $$P_{k}=\left(I-K_{k}\;H\right)\;P_{k}^{-}$$

where $K_{k}$ is denoted as the Kalman gain, which provides a weighting method between the actual measurement $z_{k}$ and their prediction $H\;{\hat{x}}_{k}^{-}$. It is worth noticing that covariance matrices *P*, *W* and *V* are semi-positive definite. While the basic KF filter provides a suitable solution for the estimation of linear equation systems, its applicability is generally restricted in wider scenarios. In the automotive field, vehicle dynamics models are usually strongly nonlinear, leading to the frequent adoption of the first-order extended Kalman filter (FO-EKF).

### 2.1. Extended Kalman Filter

For nonlinear stochastic difference equations, the equations can be rewritten as follows (Equations (8) and (9)):

(8) $$x_{k}=f\left(x_{k-1},u_{k-1},w_{k-1}\right)$$

(9) $$z_{k}=h\left(x_{k},v_{k}\right)$$

The generic function *f* relates the state at the previous time step k−1 to the state at the current time step k. The main concept of this formulation is to linearize the system, at each time step, around the estimated state of the system at the previous time step, by employing the Jacobian matrices (Equations (10)–(13)):

(10) $$A_{k[ij]}=\frac{\partial f[i]}{\partial x[j]}\left({\hat{x}}_{k-1},u_{k-1},0\right)$$

(11) $$W_{k[ij]}=\frac{\partial f[i]}{\partial w[j]}\left({\hat{x}}_{k-1},u_{k-1},0\right)$$

(12) $$H_{k[ij]}=\frac{\partial h[i]}{\partial x[j]}\left({\hat{x}}_{k}^{-},0\right)$$

(13) $$V_{k[ij]}=\frac{\partial h[i]}{\partial v[j]}\left({\hat{x}}_{k}^{-},0\right)$$

where $A_{k[ij]}$, $W_{k[ij]}$, $H_{k[ij]}$, and $V_{k[ij]}$, are the generic element of, respectively, $A_{k}$, $W_{k}$, $H_{k}$, and $V_{k}$, on row i and column j, and $f_{i}$, $h_{i}$, $x_{i}$, $v_{i}$, and $w_{i}$ represent the i-th element of, respectively, f, h, x, v, and w. Therefore, the equation contains the Jacobian matrices of the partial derivatives of the process and measurement functions with respect to the state and the noise. Time update equations (Equations (14) and (15)) are now introduced to describe the evolution of the EKF:

(14) $${\hat{x}}_{k}^{-}=f\left({\hat{x}}_{k-1},u_{k-1},0\right)$$

(15) $$P_{k}^{-}=A_{k}\;P_{k-1}\;A_{k}^{T}+W_{k}\;Q_{k-1}\;W_{k}^{T}$$

and the a posteriori equations (Equations (16)–(18)) are

(16) $$K_{k}=P_{k}^{-}\;H_{k}^{T}{\left(H_{k}\;P_{k}^{-}H_{k}^{T}+V_{k}\;R_{k}\;V_{k}^{T}\right)}^{-1}$$

(17) $${\hat{x}}_{k}={\hat{x}}_{k}^{-}+K_{k}\left(z_{k}-h\left({\hat{x}}_{k}^{-},0\right)\right)$$

(18) $$P_{k}=\left(I-K_{k}\;H_{k}\right)\;P_{k}^{-}$$

Despite the efficient solution provided by the introduction of the EKF to estimate the state of a nonlinear system, some important aspects need to be underlined. They can briefly be summarized:

- The calculation of Jacobian matrices may be computationally demanding; this problem is amplified in scenarios where partial derivatives have to be evaluated online at each time step.
- The linearization process results in accurate estimation only when the error propagation can be well approximated by a linear model.

Many studies have been proposed in order to overcome these flaws. Some of them relate to the introduction of high-order Kalman filters or more sophisticated versions of the EKF [65,69,70,71,72].

### 2.2. Iterated Extended Kalman Filter

The main concept behind the iterated extended Kalman filter (I-EKF) is acting on the linearization error by reformulating the Taylor series expansion around the a posteriori state estimate [57]. The process can be iterated multiple times; however, the majority of the possible improvement is obtained after the first relinearization, as stated in the literature. For the sake of completeness, it should be mentioned that the second-order extended Kalman filter (SO-EKF) can be employed also, and its main feature involves performing a second-order Taylor expansion of process equations. The main difference between the two approaches can be found in the iteration cycle that aims at refining the measurement update equations at generic time k. The mathematical set of equations is equal to the previously introduced FO-EKF except for the adoption of a recursive update of the state estimate using the best state estimate available [57]. See Algorithm 1.
**Algorithm 1** Iterated Extended Kalman Filter Algorithm 1:${\hat{x}}_{0}$                          ▹ Define initial state 2:$P_{0}$                     ▹ Define initial state covariance 3:**for**k=1 to *T* **do** 4:   ${\hat{x}}_{k}^{-}=f\left({\hat{x}}_{k-1},u_{k-1},0\right)$                ▹ a priori state estimate 5:   $P_{k}^{-}=A_{k-1}P_{k-1}A_{k-1}^{T}+Q$        ▹ a priori state estimate covariance 6:   ${\hat{x}}_{k,1}={\hat{x}}_{k}^{-}$ 7:   **for** i=1 to *N* **do** 8:     $z_{k,i}=h\left({\hat{x}}_{k,i},u_{k},0\right)-H_{k,i}\left({\hat{x}}_{k}^{-}-{\hat{x}}_{k,i}\right)$   ▹ a priori measurement estimate 9:     $K_{k,i}=P_{k}^{-}H_{k,i}^{T}{\left(H_{k,i}P_{k}^{-}H_{k,i}^{T}+R\right)}^{-1}$             ▹ Kalman gain10:     ${\hat{x}}_{k,i+1}={\hat{x}}_{k}^{-}+K_{k,i}\left(y_{k}-z_{k,i}\right)$        ▹ a posteriori state estimate11:     $P_{k,i+1}=\left(I-K_{k,i}H_{k,i}\right)P_{k}^{-}$      ▹ a posteriori state estimate covariance12:   **end for**13:**end for**

### 2.3. Simply Unscented Kalman Filter

An unscented Kalman filter (UFK) provides an alternative approach to propagate state and error covariance during a nonlinear process, applying the transformation on single points rather than on a Gaussian distribution. The algorithm performs the nonlinear transformation on a set of so-called sigma points (Equations (19)–(21)), whose sample pdf well approximates the pdf of the instantaneous state estimate. The described approach ensures robustness to evaluate high-order nonlinear state evolution, also employing a reduced number of sigma points. If *x* is a [nx1] vector that is transformed by the nonlinear function y=f(x), the 2n sigma points are

(19) $$x^{\left(i\right)}=\bar{x}+{\tilde{x}}^{\left(i\right)}\;\;\;\;i=1,\ldots \ldots ,2n$$

(20) $${\tilde{x}}^{\left(i\right)}={\left(\sqrt{nP_{xx}}\right)}_{i}^{T}\;\;\;\;i=1,\ldots \ldots ,n$$

(21) $${\tilde{x}}^{\left(n+i\right)}=-{\left(\sqrt{nP_{xx}}\right)}_{i}^{T}\;\;\;\;i=1,\ldots \ldots ,n$$

where $\bar{x}$ is the mean of x and $P_{xx}$ is its covariance; $\sqrt{nP_{xx}}$ is the matrix square root of $nP_{xx}$ and ${\left(\sqrt{nP_{xx}}\right)}_{i}$ is the i-th row of $\sqrt{nP_{xx}}$. To evaluate a matrix square root, the Cholesky factorization can be applied. Furthermore, applying the nonlinear function to each individual sigma point, the transformed entities are computed as

(22) $$y^{\left(i\right)}=f\left(x^{\left(i\right)}\right)\;\;\;\;i=1,\ldots ,2n$$

the approximated mean of *y* is given by

(23) $$\bar{y}=\frac{1}{2n}\sum_{i=1}^{2n}y^{\left(i\right)}$$

It has been demonstrated that the computed mean approximates the true mean and covariance of *y* up to the third order, whereas the linearization only matches the true mean of *y* up to the first order.

(24) $$P_{yy}=\frac{1}{2n}\sum_{i=1}^{2n}\left(f\left(x^{\left(i\right)}\right)-\bar{y}\right){\left(f\left(x^{\left(i\right)}\right)-\bar{y}\right)}^{T}$$

In general, it can be said that the UFKs propagate the mean and the covariance of the sigma points using systems of nonlinear equations, and the a priori state estimate is the weighted mean of them. This also applies to the predicted measurement that can be computed using all measurement equations for each propagated sigma point, and the predicted measurement vector is the weighted mean of them. Unlike EKFs, in UFKs, there is the introduction of a cross-covariance matrix. The simplest UKF, known as simply unscented Kalman filter (S-UKF), is characterized by the use of 2n sigma points and equal weights.

### 2.4. General Unscented Kalman Filter

The selection of sigma points and weight becomes crucial to classify different observers; in [73,74], it is shown that the same order of mean and covariance estimation accuracy can be obtained by choosing 2n+1 sigma points (Equations (25)–(28)). This type of UKF is referred as a general unscented Kalman filter (G-UKF).

(25) $$x^{\left(0\right)}=\bar{x}$$

(26) $$x^{\left(i\right)}=\bar{x}+{\tilde{x}}^{\left(i\right)}\;\;\;\;i=1,\ldots \ldots ,2n$$

(27) $${\tilde{x}}^{\left(i\right)}={\left(\sqrt{\left(n+k\right)P_{xx}}\right)}_{i}^{T}\;\;\;\;i=1,\ldots \ldots ,n$$

(28) $${\tilde{x}}^{\left(n+i\right)}=-{\left(\sqrt{\left(n+k\right)P_{xx}}\right)}_{i}^{T}\;\;\;\;i=1,\ldots \ldots ,n$$

Another feature is the use of different weights (Equations (29) and (30)):

(29) $$W^{\left(0\right)}=\frac{k}{n+k}$$

(30) $$W^{\left(i\right)}=\frac{1}{2(n+k)}\;\;\;\;i=1,\ldots \ldots ,2n$$

In the weight’s definition, the introduction of the parameter *k* is useful to decrease high-order errors in mean and covariance propagation. Indeed, if *x* is normally distributed, then choosing k=3−n results in a minimization of the fourth-order terms error. It is, then, possible to apply the nonlinear transformation to each sigma point (Equations (31) and (32)):

(31) $$\bar{y}=\sum_{i=0}^{2n}W^{\left(i\right)}y^{\left(i\right)}$$

(32) $$P_{yy}=\sum_{i=0}^{2n}W^{\left(i\right)}\left(f\left(x^{\left(i\right)}\right)-\bar{y}\right){\left(f\left(x^{\left(i\right)}\right)-\bar{y}\right)}^{T}$$

It is worth noticing that the S-UKF formulation can easily be obtained by choosing k=0, resulting in 2n sigma points and equal weights. Furthermore, it should be mentioned that other variations of the same algorithm can be found in the literature; the key concept is modeling the distribution of sigma points around the mean state value, employing parameters (α, *k*), while the introduction of the β value relates to the definition of weights of the transformed points.

### 2.5. Simplex Unscented Kalman Filter

Another set of sigma points and weight factors can be introduced; therefore, choosing n+1 sigma points for *x* (*x* has *n* elements) results in the minimum number of sigma points which gives the same order of estimation accuracy. This observer aims to reduce computational burden by acting on the number of sigma points without losing performance. The algorithm presented in [74] deals with n+2 sigma points, but the number can be reduced to n+1 by choosing one weight to be zero. Although this filter can be helpful for high dimension problems, it is shown that the ratio of $W^{\left(n\right)}$ to $W^{\left(1\right)}$ is $2^{n-2}$ (same for $\sigma_{i}^{n}$), resulting in numerical problems as the dimension of the state increases.

### 2.6. Spherical Unscented Kalman Filter

The proposed UKF aims to rearrange the sigma points of the S-UKF to obtain better numerical stability. The filter is referred as a spherical unscented Kalman filter (SPHE-UKF), and its main features are the equal weight factors and the ratio formulation (Equation (33)):

(33) $$\frac{n}{\sqrt{n\left(n+1\right)W^{\left(i\right)}}}/\frac{1}{\sqrt{n\left(n+1\right)W^{\left(1\right)}}}=n$$

In general, all UFKs have a great advantage, as they do not require Jacobian or Hessian computation, but the main obstacle is the state covariance matrices that must be positive semidefinite to obtain real matrices after Cholesky decomposition. Furthermore, sigma points’ evaluation is required at each time step, leading to computational cost.

## 3. State Estimation Architecture

### 3.1. Model Equations

Vehicle modeling evaluates three groups of equations: kinematic (congruence) equations, equilibrium equations, and tire constitutive equations. The definition of the set of equations depends on the vehicle model adopted. The commonly used vehicle models are the double-track and the single-track models, which differ from each other in the assumptions underlying the model. The advantage of using one or the other depends on the results needed and the kind of precision requested. Generally, the single-track model is enough to describe a vehicle’s behavior, but due to the main assumption that consists of neglecting the roll motion, it is impossible to describe the vertical dynamics. Regarding this paper, since in the following discussions in-plane dynamics is described, the single-track assumptions allow us to evaluate all the vehicle equations needed. The single-track model consists of only one wheel per axle. The Ackermann coefficient is set equal to zero. If the static toe is null, due to this hypothesis, the steering angles of the front wheels are the same, so the wheels are characterized by parallel steering. Considering $\delta_{1}$ as the front axle steering angle (Equations (34) and (35)),

(34) $$\delta_{11}=\delta_{12}=\delta_{1}$$

(35) $$\delta_{21}=\delta_{22}=\delta_{2}$$

where $\delta_{11}$ and $\delta_{12}$ are the steering angles of the left and right front tires in a double-track model, and $\delta_{21}$ and $\delta_{22}$ are related to the rear tires.

**Figure 2:**
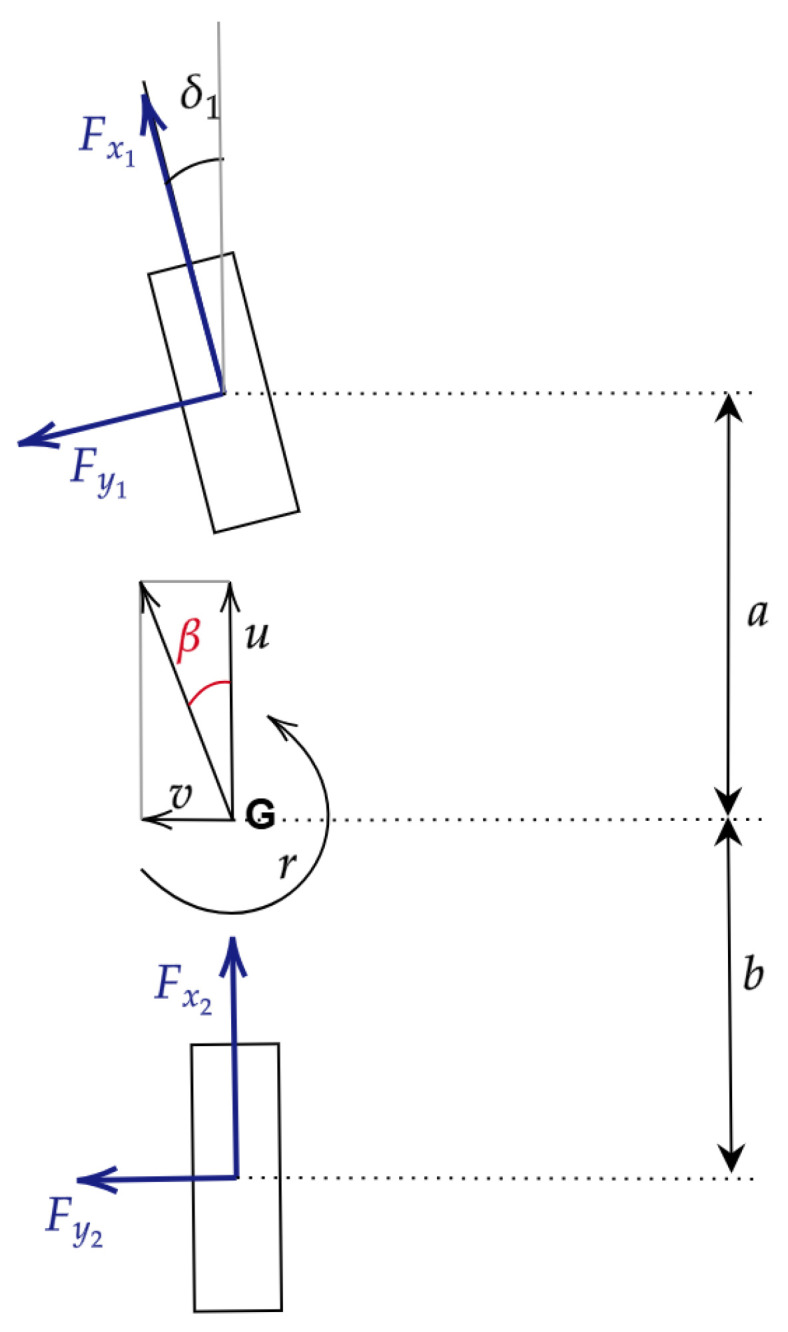
Single-track vehicle model basic scheme.

Considering Figure 2, it is possible to extrapolate the kinematics equations (Equations (36)–(39)):

(36) $$V_{x_{1}}=u\;cos\left(\delta_{1}\right)+\left(v+ra_{1}\right)sin\left(\delta_{1}\right)$$

(37) $$V_{y_{1}}=\left(v+ra_{1}\right)cos\left(\delta_{1}\right)-u\;sin\left(\delta_{1}\right)$$

(38) $$V_{x_{2}}=u$$

(39) $$V_{y_{2}}=\left(v-ra_{2}\right)$$

where x indicates the longitudinal axis of the vehicle and y indicates the lateral axis so that Vx and Vy represent the longitudinal and lateral velocities. u, v, and r are, respectively, the longitudinal, the lateral, and the yaw velocities (rate) at the center of gravity. Finally, $a_{1}$ and $a_{2}$ are, respectively, the front and rear wheelbase. Condensing two wheels per axle into a single one, coherently with the hypothesis of the single-track vehicle model, leads to the following equations (Equations (40) and (41)) to calculate tire slip angles and slip ratios, which are provided as input to the tire model, which is going to be described:

(40) $$\alpha_{j}=arctan\left(\frac{V_{y_{j}}}{\left|V_{x_{j}}\right|}\right)$$

(41) $$k_{j}=-\frac{V_{x_{j}}-\omega_{j}R_{r_{j}}}{\left|V_{x_{j}}\right|}$$

Substituting the terms of the slips equations (Equations (42)–(45)) with the ones evaluated with the kinematics equations,

(42) $$\alpha_{1}=arctan\left(\frac{\left(v+ra_{1}\right)cos\delta_{1}-usin\left(\delta_{1}\right)}{\left|ucos\left(\delta_{1}\right)+\left(v+ra_{1}\right)sin\left(\delta_{1}\right)\right|}\right)$$

(43) $$k_{1}=\frac{\omega_{1}R_{r_{1}}-ucos\left(\delta_{1}\right)+\left(v+ra_{1}\right)sin(\delta_{1})}{ucos\left(\delta_{1}\right)+\left(v+ra_{1}\right)sin\left(\delta_{1}\right)}$$

(44) $$\alpha_{2}=arctan\left(\frac{\left(v-ra_{2}\right)}{\left|u\right|}\right)$$

(45) $$k_{2}=-\frac{\omega_{2}R_{r_{2}}-u}{u}$$

Finally, the equilibrium equations can be written as follows (Equations (46)–(48)), indicating the longitudinal and lateral velocities derivative as $\dot{u}$ and $\dot{v}$ and the yaw rate derivative as $\dot{r}$:

(46) $$m(\dot{u}-vr)=X$$

(47) $$m(\dot{v}+ur)=Y$$

(48) $$J_{z}\dot{r}=N$$

where

(49) $$X=F_{x_{1}}cos\left(\delta_{1}\right)-F_{y_{1}}sin\left(\delta_{1}\right)+F_{x_{2}}$$

(50) $$Y=F_{y_{1}}cos\left(\delta_{1}\right)-F_{x_{1}}sin\left(\delta_{1}\right)+F_{y_{2}}$$

(51) $$N=F_{y_{1}}a_{1}cos\left(\delta_{1}\right)-F_{x_{1}}a_{1}sin\left(\delta_{1}\right)+F_{y_{2}}a_{2}$$

In the previous equations (Equations (49)–(51)), $F_{x_{j}}$, with j = 1,2, represents the longitudinal forces on each axle, $F_{y_{j}}$, with j = 1, 2, represents the lateral forces on each axle, X and Y represent the net forces, respectively, on the longitudinal and lateral direction, and N represents the net momentum around the z-axis. Vehicle quantities are described in Table 1.

Forces appearing at the equilibrium equations are calculated by a simplified Pacejka Magic Formula (MF) tire model [48,49,75,76]. This formulation takes into account only macroparameters, and it has been preferred among other more computationally demanding models [77,78,79,80], which require a high-cost and complex experimental routine to identify the parameters [81,82,83,84,85,86,87,88]. On the other hand, it has been preferred to other simplified approaches, which do not reproduce the nonlinear tire behavior at high slip values [48,89].

(52) $$F_{0}=D\;sin\left(C\;arctan\left[B\;x_{s}-E\left(B\;x_{s}-arctan\left(B\;x_{s}\right)\right)\right]\right)+S_{v}$$

where

(53) $$x_{s}=X_{s}+S_{h}$$

The formulation (Equations (52) and (53)) is the same for the calculation of longitudinal and lateral forces, while the independent variable $X_{s}$, respectively, identifies the tire slip ratio or slip angle, which have been previously mentioned. The described formulation refers to pure condition $F_{0}$. The parameters are now introduced:

- B: stiffness factor;
- C: shape factor;
- D: peak value;
- E: curvature factor;
- $S_{v}$, $S_{h}$: shifts from Cartesian axes center.

Furthermore, a vertical load dependence (Equation (54)) is adopted for the peak value:

(54) $$D=D\left(F_{z}\right)=\mu \;F_{z}$$

The MF is also suitable for combined slip cases where the introduction of the Hill function (*G*) is needed (Equations (55)–(57)):

(55) $$G=\frac{cos(C_{c}\;arctan\left[B_{c}\;x_{c}-E_{c}\left(B_{c}\;x_{c}-arctan\left(B_{c}\;x_{c}\right)\right)\right])}{cos(C_{c}\;arctan\left[B_{c}\;S_{H_{c}}-E_{c}\left(B_{c}\;S_{H_{c}}-arctan\left(B_{c}\;S_{H_{c}}\right)\right)\right])}$$

where

(56) $$x_{c}=X_{c}+S_{H_{c}}$$

The combined tire force is then evaluated as

(57) $$F_{c}=F_{0}\;G$$

It can be noticed that the two independent variables $X_{s}$ and $X_{c}$ assume the value of the tire slip angle and slip ratio if employed in lateral force evaluation, while their values are exchanged in longitudinal force formulation.

### 3.2. Filter Design

A common structure is adopted in designing the different model-based observers. The general structure of state variable vector is

(58) $$x_{K}={\left[u_{k},v_{k},r_{k}\right]}^{T}$$

The sideslip angle is then evaluated as $\beta =arctan(\frac{v}{u})$.

### 3.3. Measurement Equations

Measurement equations are needed to evaluate the estimate $z_{k}$ at time step *k* knowing the a priori state estimate and the additional input values $u_{k}$. In general, they can be written as

(59) $$z_{k}=h\left({\hat{x}}_{k}^{-},u_{k}\right)+v_{k}$$

For this work, a set of five measurements was chosen, and the criteria were taking advantage of low-end sensors such as encoders for sensing the wheel speed and IMU for accelerations. The acquired signal vector is now presented:

(60) $$y_{k}=\left[\omega_{1,1_{k}}^{ENCODER},\omega_{1,2_{k}}^{ENCODER},r_{k}^{IMU},ax_{k}^{IMU},ay_{k}^{IMU}\right]$$

The estimated measurement vector will then be

(61) $$z_{k}=\left[\omega_{1,1_{k}},\omega_{1,2_{k}},r_{k},ax_{k},ay_{k}\right]$$

*h* functions are able to estimate $y_{k}$ using typical vehicle dynamics equations. The yaw rate is defined as both the state and measurement variable; therefore, the h() function will be an identity.

(62) $$ax_{k}=X/m$$

(63) $$ay_{k}=Y/m$$

(64) $$r_{k}=r_{k}$$

(65) $$\omega_{11_{k}}=Vx_{11_{k}}/R_{r_{1}}$$

(66) $$\omega_{12_{k}}=Vx_{12_{k}}/R_{r_{1}}$$

Since the wheel angular velocities are available at the vehicle corners, the wheel linear velocities $Vx_{11_{k}}$$Vx_{12_{k}}$ appearing at the previous equations (Equations (62)–(66)) have to be obtained by translating the vehicle velocities from the center of gravity to the hub locations coherently with a double-track formulation [90]. Moreover, they have to be rotated to account for the wheel steering angle. In addition, for the filter to properly work, input signals need to be defined. For this application, the steering wheel angle, wheels’ rotational velocity and longitudinal acceleration are used.

### 3.4. QR Calibration

During the description of KF-based filters’ variables such as process noise and measurement noise, respectively, *w* and *v* were introduced. It can be said that the process noise represents the discrepancy between the target state value and the model’s a priori estimate; on the other hand, the measurement noise is generally related to the employed sensor’s accuracy. The previously introduced variables *w* and *v* are considered mutually independent, white and normally distributed (Equations (67) and (68)):

(67) p(w)∼N(0,Q)

(68) p(v)∼N(0,R)

It can be said that model errors are more systematic than measurement errors. *Q* and *R* are defined as process and measurement noise covariance matrices, whose definition is fundamental to reach the best possible accuracy and robustness of the estimation. Their definition is often a hard task. Because sensor errors are likely to be uncorrelated, *R* will result in a diagonal matrix, while for *Q*, manual tuning is practically impossible. In this work, *Q* and *R* are determined through the so-called “Maximizing the Joint Likelihood’’ algorithm [91], which is based on an offline calculation of the errors made by the model to update the state, starting from the target state defined at the previous instant (Equations (69) and (70)). The target state $x_{k}$ has been provided by the S-motion readings, and the errors’ covariance has been calculated on the entire data collection (*N* is the number of samples).

(69) $$Q=\frac{1}{N}\sum_{i=1}^{N}\left(x_{k}-f\left(x_{k-1},u_{k-1}\right)\right){\left(x_{k}-f\left(x_{k-1},u_{k-1}\right)\right)}^{T}$$

(70) $$R=\frac{1}{N}\sum_{i=1}^{N}\left(y_{k}-h\left(x_{k}\right)\right){\left(y_{k}-h\left(x_{k}\right)\right)}^{T}$$

This procedure ensures an objective comparison between the examined filters, because the Q and R matrices have been calculated through a deterministic method and their values are only related to the model and sensor accuracy with respect to the target data.

## 4. Experimental Campaign

To collect the required data for calibration and validation of the proposed filters, a testing procedure was conducted with a test vehicle. Initially, all the relevant vehicle parameters were collected as presented in Table 2.

The required vehicle signals were collected by utilizing a set of low and high-end sensors. The required measurements for the estimator filters were obtained through common affordable vehicle sensors such as an inertial measurement unit (IMU) and provided by the standard controller area network (CAN). On the other hand, an optical sensor was also installed to obtain reliable measurements of the vehicle’s longitudinal and lateral speeds, which are both crucial to the accurate calculation of the actual sideslip angle. The complete list of signals and respective sensors used for this study is presented in Table 3.

**Figure 3:**
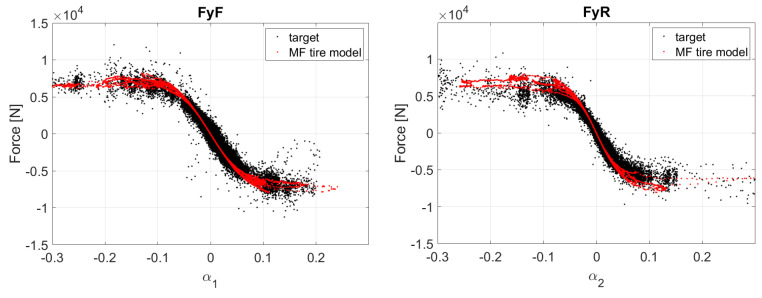
Tire model calibration results.

To characterize the tire–road interaction, a specific testing procedure known as TRICK [95] was conducted. The driver was instructed to employ four different driving styles (*Gentleman*, *Pure Interaction*, *Max Performance* and *Sliding*), which attempt to stress the tires in different conditions, as presented in Table 4. The obtained data, along with the previously mentioned vehicle parameters, were then fed into an inverse model to calculate the relevant tire kinematic and dynamic quantities such as slips and forces (Figure 3).

The tire model-fitting process followed a classic approach that can be split into different phases. Initially, a preprocessing routine was applied to remove outliers and collect the relevant samples for the calibration procedure. A reference nominal load was selected for the fitting process and was then used as a reference to start calibrating the force–slip curves in pure lateral and longitudinal conditions. The admissible values of slip ratio and slip angle were limited to a small range for the lateral and longitudinal models, respectively, to exclude any combined interaction from the fitting process. Once the fitting at nominal vertical loads was satisfactory, the load dependency characteristic was calibrated by selecting vertical load ranges below and above the nominal condition. As a simplified Magic Formula was employed for the estimators’ tire model, no other dependencies such as camber, pressure or temperature were taken into account. The final step of the calibration procedure focused on identifying the combined interaction, fitting the force–slip curve for combined values of slip angle and slip ratio.

## 5. Results

In this section, a comparative analysis of different filters is introduced. To underline specific features, different runs were properly chosen. They involve both short and long acquisition in terms of time and different conditions of lateral excitation, which are reached through combining the previously introduced driving styles (Table 5). All the simulations were performed using the MATLAB R2023b environment on a PC employing WINDOWS 11 with a 13th Gen Intel(R) Core(TM) i7-1370P @1.90 GHz (Santa Clara, CA, USA).

**Figure 4:**
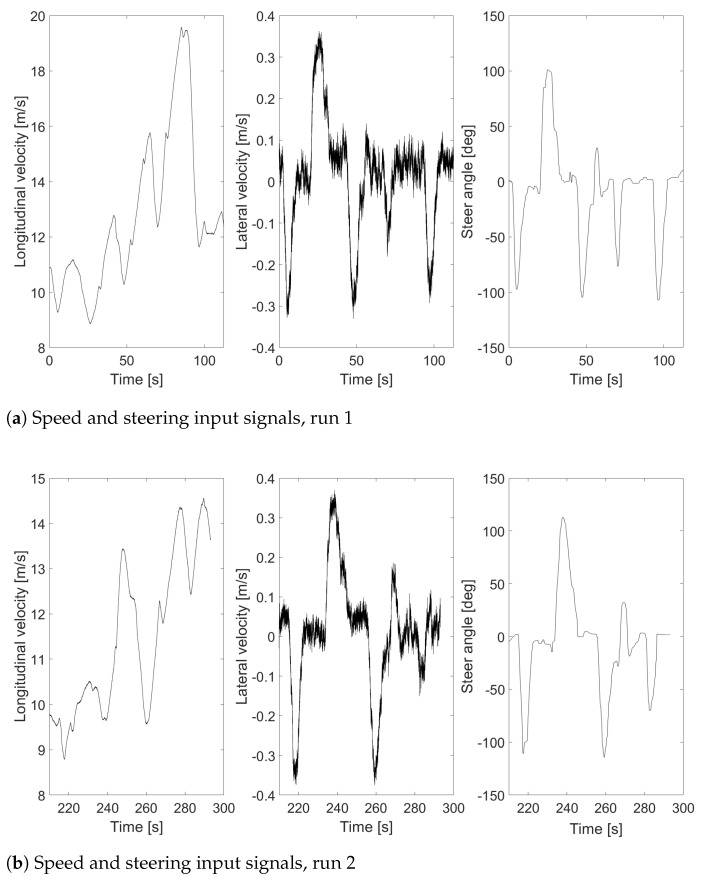

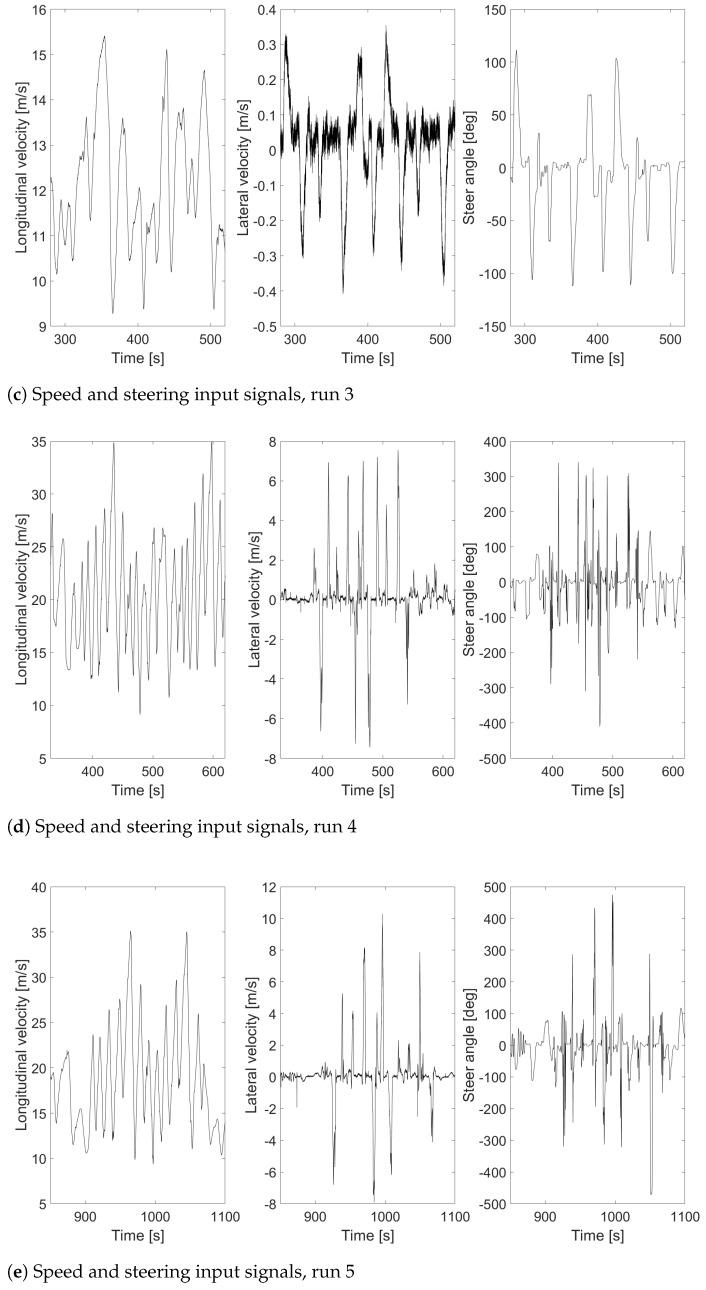

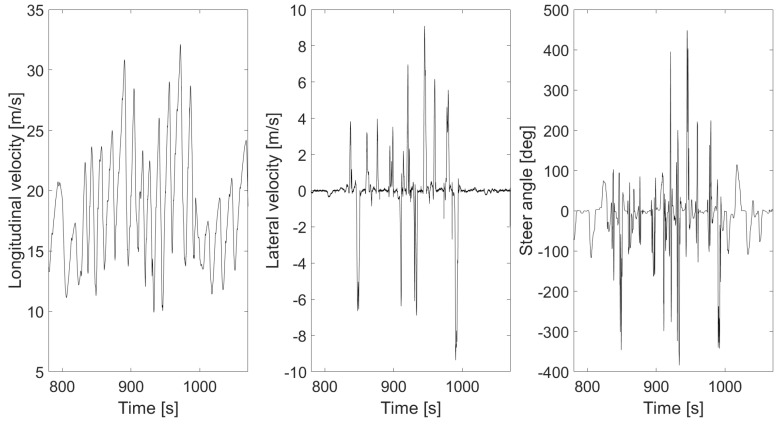
Speed and steering input signals: (**a**) run 1, (**b**) run 2, (**c**) run 3, (**d**) run 4, (**e**) run 5, (**f**) run 6.

To provide the reader with more accurate details on the different runs, some major input signals are described in Figure 4.

**Figure 5:**
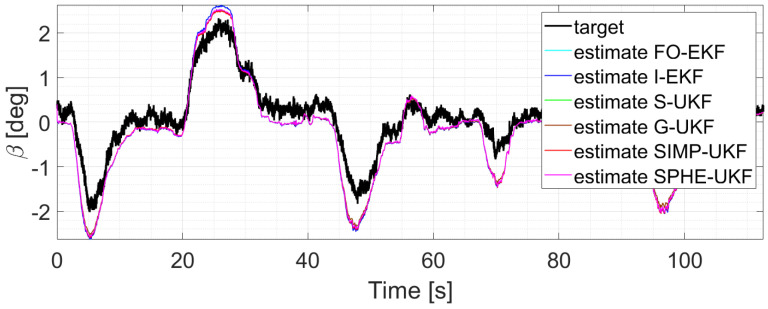
Comparison of all the observers, run 1.

**Figure 6:**
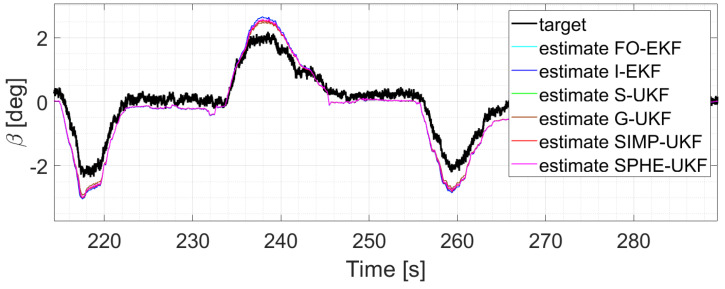
Comparison of all the observers, run 2.

**Figure 7:**
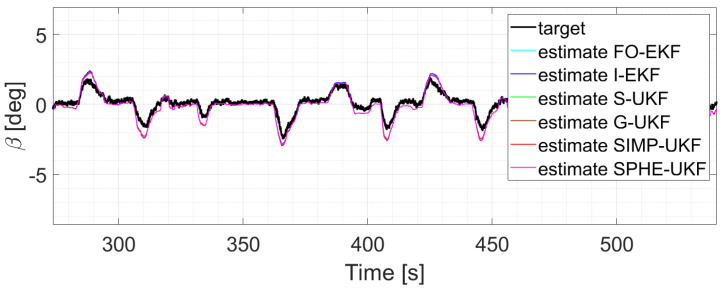
Comparison of all the observers, run 3.

The first three runs are initially analyzed, and the performance of the six observers is almost the same as they explore relatively reduced levels of β values (Figure 5, Figure 6 and Figure 7).

**Figure 8:**
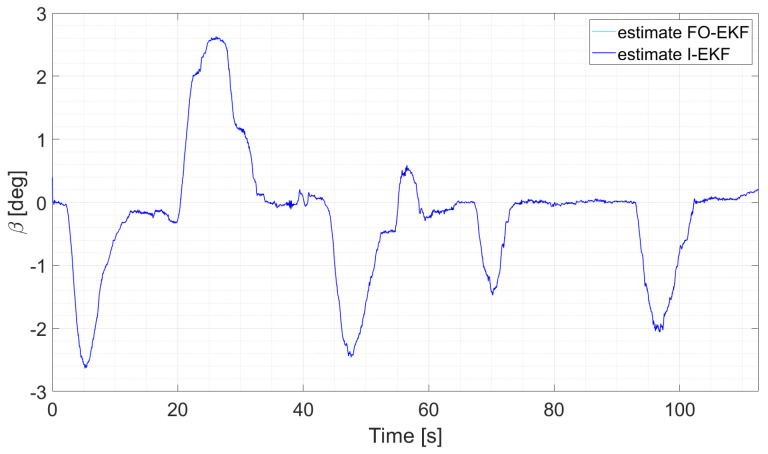
Comparison of EKFs, run 1.

**Figure 9:**
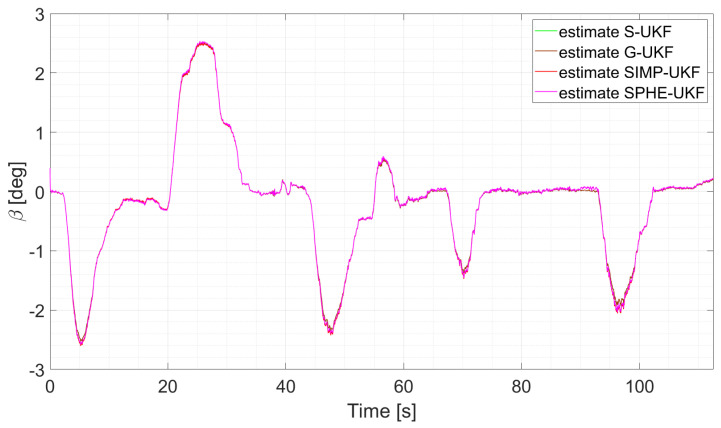
Comparison of UKFs, run 1.

In particular, considering run 1 (equal consideration can be made for run 2 and run 3), the two EKFs show the same level of accuracy, and the same result can be observed when comparing UKFs (Figure 8 and Figure 9).

**Figure 10:**
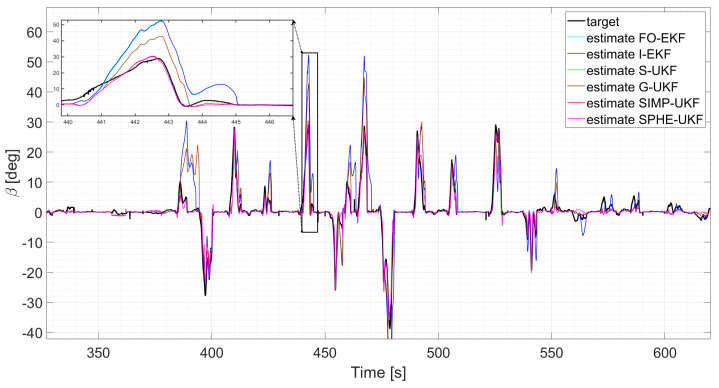
Comparison of all the observers, run 4.

**Figure 11:**
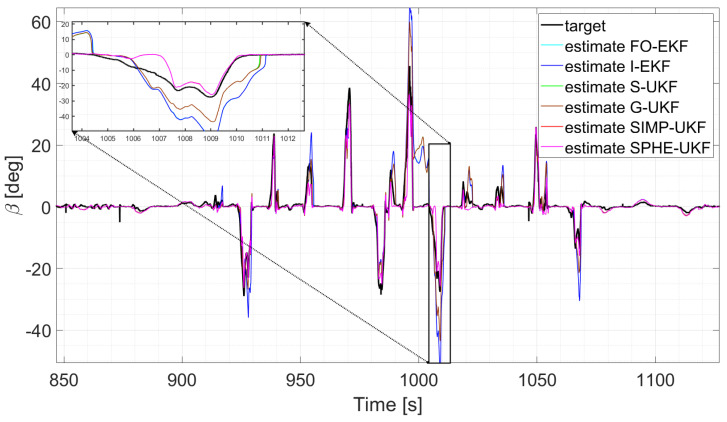
Comparison of all the observers, run 5.

Moreover, the last three runs are discussed; they exploit an increasing number of laps and driving styles, exploring both high and low levels of lateral excitement (Figure 10 and Figure 11). Considering, for instance, run six, as its results can be generalized for the other two, a comparative analysis shows that

**Figure 12:**
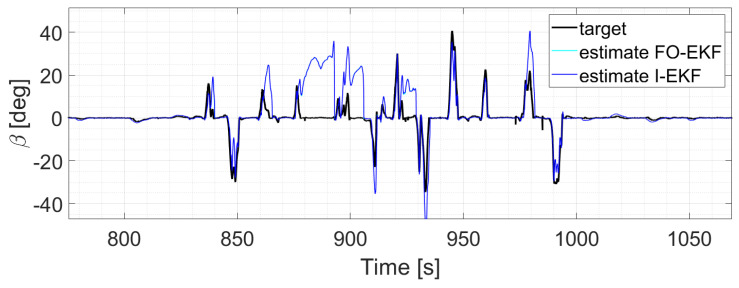
Comparison of EKFs, run 6.

**Figure 13:**
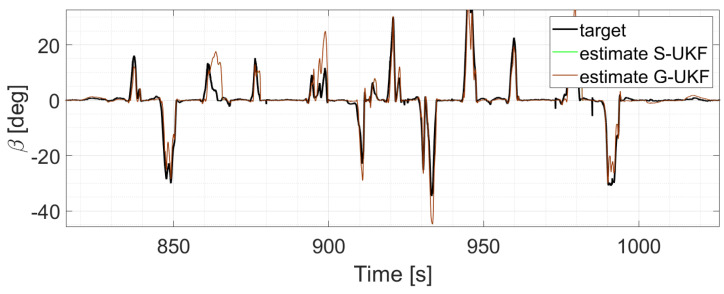
Comparison of S-UKF vs. G-UKF, run 6.

**Figure 14:**
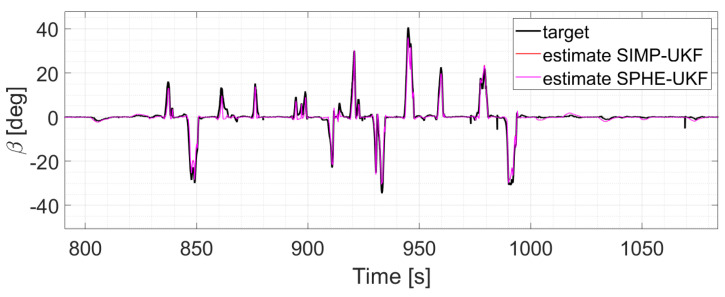
Comparison of SIMP-UKF vs. SPHE-UKF, run 6.

**Figure 15:**
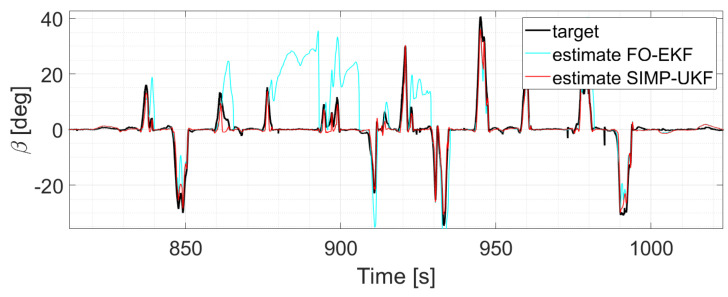
Comparison of EKF vs. SIMP-UKF, run 6.

**Figure 16:**
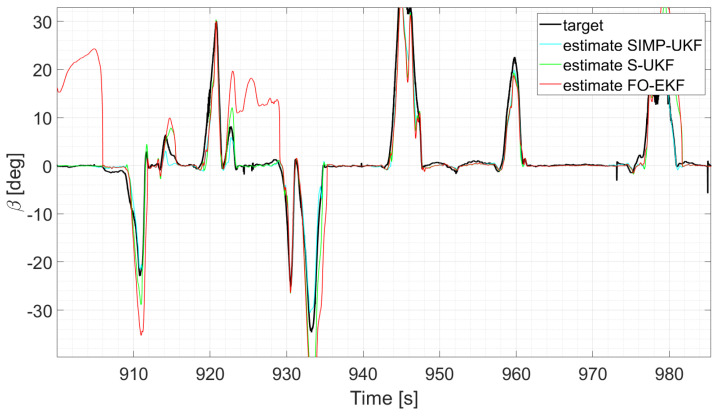
Comparison of all the observers, run 6.

- The two EKFs perform equally (Figure 12);
- S-UKF and G-UKF show comparable results (Figure 13);
- SIMP-UKF and SPHE-UKF have almost the same estimation accuracy (Figure 14);
- In general, SIMP/SPHE-UFKs perform the best out of all the observers; while S-UKF and G-UKF are more precise than the two EKFs (Figure 15 and Figure 16).

It is worth noticing how the performance of the different observers changes as the run explores the various levels of lateral excitation. Indeed, for lower values of sideslip angle, the performances are comparable, while on the other hand, the estimation error of the EKFs tends to increase. This can be due to the highly nonlinear conditions reached during those specific runs, which results in the worse performance shown by the two EKF filters.

**Figure 17:**
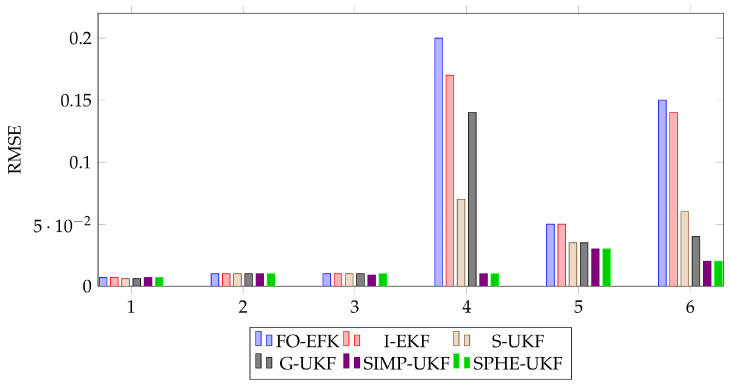
Visual representation of RMSE comparison between all the observers on each run.

To provide the reader with a more general overview of the estimation accuracy reached through the simulations, the root mean square error (RMSE) of the estimated sideslip angle has been evaluated, and it is used as a term of comparison (Table 6, Figure 17).

Furthermore, a comparison analysis of the computational time needed for the different filters to work is presented. Each observer has been executed multiple times for each run to obtain the execution time average values reported in Table 7. To summarize the results of this analysis, the mean computational time has been calculated for each filter, and it has been reported in the last column of Table 7. When comparing the two EKFs, it is obvious that more iterations lead to additional time needed (I-EKF). SIMP-UKF and SPHE-UKF are the fastest among the other UFKs, and this is due to the lower number of sigma points. Indeed, it can be said that the G-UFK is slower than the S-UKF, because not only does it have a larger number of sigma points, but also different weights are applied to them.

## 6. Conclusions

In the current article, a qualitative and quantitative comparison has been carried out on the estimation results of KF-based state observers with variable accuracy and complexity to perform real-time estimation of the vehicle sideslip angle. Since a dynamic approach has been performed, employing a Magic Formula tire model, the calibration procedure required an outdoor experimental campaign to identify the parameters during the pure and combined interaction. The performed on-track experimental campaign also provided the on-board measurements acquired to validate the estimation algorithm, including the low-end sensors useful within the analytical estimation process and the accurate high-end sensors’ signals that provide the optimal target for the results’ comparison.

Differing maneuvers have been performed to investigate the performance of the filters in a wide operating domain and to verify the errors due to linearization when the vehicle works in critical driving conditions and its dynamics become strongly nonlinear. To account for the necessity of employing the algorithm in on-board scenarios, also the computational cost of the differing filters has been evaluated by comparing the respective computational time taken to perform a simulation of the online estimation.

As shown in the Results, within the linear working range, characterized by small sideslip angle values and negligible high-order transient dynamics, the improvements provided by high-complexity solutions compared with the simple FO-EKF are not sensitive enough to justify their employment. However, on the other hand, nonlinear conditions with higher values of sideslip angle lead to increasing estimation error when a linear formulation is applied to propagate error covariance and a Gaussian distribution around the previous state estimate is supposed. This occurs also when the estimator employs a redundant iterative procedure that increases the computational effort (I-EKF).

The UKF-based observer showed great accuracy also in this range, demonstrating their suitability in all the explored operating conditions; furthermore, for this estimation architecture, most detailed formulations do not ensure increasing accuracy, as evidenced by the comparisons with the experimental target. This justifies the employment of the version of the UKF, SIMP-UKF, which provides the optimal balance in terms of computational effort and estimation robustness.

Based on these results, further developments will include the development of a hybrid observer to optimize the estimation performance, which would employ an EKF formulation, switching on the nonlinear UKF implementation toward nonlinear vehicle driving situations. However, to avoid a too complex formulation, the dynamic-based EKF could be integrated into a fuzzy-logic architecture, working in feedback with a kinematic estimator, which is more accurate in nonlinear conditions and during transient maneuvers. In the end, to extend this study to a less customized context, the sensitivity analysis will be carried out by employing experimental data acquired on-board different vehicles.

## Figures and Tables

**Table 1:** Vehicle quantities.

Quantity Name	Symbol	Units
Vehicle mass	*m*	kg
Inertia moment around z-axis	$J_{z}$	$kg\;m^{2}$
Steering angle	$\delta_{j}$	rad
Front wheelbase	$a_{1}$	*m*
Rear wheelbase	$a_{2}$	*m*
Longitudinal CoG speed	*u*	m/s
Longitudinal CoG acceleration	$\dot{u}$	$m/s^{2}$
Lateral CoG speed	*v*	m/s
Lateral CoG acceleration	$\dot{v}$	$m/s^{2}$
Vehicle yaw rate	*r*	rad/s
Vehicle yaw acceleration	$\dot{r}$	$rad/s^{2}$
Lateral slip angle	$\alpha_{j}$	rad
Longitudinal slip ratio	$k_{j}$	-
Axle longitudinal force	$F_{x_{j}}$	N
Axle lateral force	$F_{y_{j}}$	N
Net longitudinal force	*X*	*N*
Net lateral force	*Y*	*N*
Net momentum around z axis	*N*	*N*
Axle angular velocity	$\omega_{j}$	rad/s
Rolling radius	$R_{r_{j}}$	*m*

**Table 2:** Vehicle parameters.

Parameter Name	Value	Units
Vehicle Mass	1197.5	kg
Unsprung Mass	200	kg
Wheelbase	2.31	*m*
Front Wheelbase	1.088	*m*
Front Track Width	1.495	*m*
Rear Track Width	1.505	*m*
CG Height	0.489	*m*
Front Roll Center Height	0.0767	*m*
Rear Roll Center Height	0.2084	*m*
Z Inertia Moment	950	$kg\;m^{2}$
Nominal Steer Ratio	14.2	-

**Table 3:** Vehicle sensors and respective accuracy.

Signal	Device	Accuracy/Sensitivity
Longitudinal Velocity	S-Motion [92]	<±0.2%
Lateral Velocity		Range: up to 400 km/h
Longitudinal Acceleration	OxTS 3000 [93]	Bias stability: 2 μg
Lateral Acceleration		Range: ±10 g
Vertical Acceleration		Bias stability: $2^{\circ }/$h
Yaw Rate		Range: $\pm 100^{\circ }/$s
Steer Angle	Steering Wheel Sensor (CAN [94])	Resolution: $0.1^{\circ }$ Range: $\pm 780^{\circ }$
Wheel Speeds (FL, FR, RL, RR)	Wheel Speed Encoder (CAN [94])	Timing accuracy: 2% Range: 0 to 2500 Hz

**Table 4:** Driving styles’ description.

Driving Style	Description	Goal
Gentleman	Driving at a relatively high pace around the track	Characterize pure and combined linear tire range
Max Performance	Driving as fast as possible around the track	Characterize pure and combined peak tire performance
Pure Interaction	Avoiding combined tire–road interaction by separate use of throttle/brake and steering	Characterize the tire’s pure interaction curves
Sliding	Forcing the tire to work in the frictional region	Characterize the “over the peak’’ region of the interaction curves

**Table 5:** Breakdown of each run.

Run	Driving Styles	N. Lap	Duration [s]
1	Gentleman	1	120
2	Gentleman	1	80
3	Gentleman	2	250
4	Pure Interaction	1	300
	Sliding Lap	2	
	Max Performance	1	
5	Gentleman	1	250
	Sliding Lap	2	
	Gentleman	1	
6	Gentleman	1	300
	Max Performance	1	
	Gentleman	1	

**Table 6:** RMSE comparison between all the observers.

	RUN					
**RMSE**	**1**	**2**	**3**	**4**	**5**	**6**
FO-EFK	0.007	0.01	0.01	0.20	0.05	0.15
I-EKF	0.007	0.01	0.01	0.17	0.05	0.14
S-UKF	0.006	0.01	0.01	0.07	0.035	0.06
G-UKF	0.006	0.01	0.01	0.14	0.035	0.04
SIMP-UKF	0.007	0.01	0.009	0.01	0.030	0.02
SPHE-UKF	0.007	0.01	0.01	0.01	0.030	0.02

**Table 7:** Comparison of time taken for each observer on the different runs.

	Mean Computational Time (Single Run) [s]						
**Observer**	**1**	**2**	**3**	**4**	**5**	**6**	**Average Computational Time [s]**
FO-EKF	2.8	2.5	6.5	7.7	6.8	8.1	5.8
I-EKF	7.2	6.4	13.2	15	13.5	15.5	11.8
S-UKF	7.6	7.4	15.1	17	15.5	17.3	13.3
G-UKF	10.2	9.6	20	23.5	19.3	23	17.6
SIMP-UKF	7.4	6.3	13.4	16.4	13.4	15.9	12.1
SPHE-UKF	7.4	6.4	13.4	16.4	13.6	16.5	12.3

## Data Availability

Data presented in this study are available on request from the corresponding author.
